# Interactions of Environmental Chemicals and Natural Products With ABC and SLC Transporters in the Digestive System of Aquatic Organisms

**DOI:** 10.3389/fphys.2021.767766

**Published:** 2022-01-13

**Authors:** Riccardo F. Romersi, Sascha C. T. Nicklisch

**Affiliations:** Department of Environmental Toxicology, University of California, Davis, Davis, CA, United States

**Keywords:** multixenobiotic resistance, intestine, ABC transporters, aquatic, **transporter**-interfering chemicals (TICs), induction, SLC transporters

## Abstract

An organism’s diet is a major route of exposure to both beneficial nutrients and toxic environmental chemicals and natural products. The uptake of dietary xenobiotics in the intestine is prevented by transporters of the Solute Carrier (SLC) and ATP Binding Cassette (ABC) family. Several environmental chemicals and natural toxins have been identified to induce expression of these defense transporters in fish and aquatic invertebrates, indicating that they are substrates and can be eliminated. However, certain environmental chemicals, termed Transporter-Interfering Chemicals or TICs, have recently been shown to bind to and inhibit fish and mammalian P-glycoprotein (ABCB1), thereby sensitizing cells to toxic chemical accumulation. If and to what extent other xenobiotic defense or nutrient uptake transporters can also be inhibited by dietary TICs is still unknown. To date, most chemical-transporter interaction studies in aquatic organisms have focused on ABC-type transporters, while molecular interactions of xenobiotics with SLC-type transporters are poorly understood. In this perspective, we summarize current advances in the identification, localization, and functional analysis of protective MXR transporters and nutrient uptake systems in the digestive system of fish and aquatic invertebrates. We collate the existing literature data on chemically induced transporter gene expression and summarize the molecular interactions of xenobiotics with these transport systems. Our review emphasizes the need for standardized assays in a broader panel of commercially important fish and seafood species to better evaluate the effects of TIC and other xenobiotic interactions with physiological substrates and MXR transporters across the aquatic ecosystem and predict possible transfer to humans through consumption.

## Introduction

Fish and other aquatic animals can be exposed to water-soluble environmental chemicals via uptake through the gills and hydrophobic xenobiotics through ingestion of contaminated food. Gills are commonly considered a part of the respiratory system of aquatic animals (Collinder et al., 2009; Carvalho, 2011; Ray and Ringø, 2014). In addition to respiration, fish gills are critical for excretion of nitrous waste, pH regulation, hormone production, and osmoregulation (Foyle et al., 2020; Zhang et al., 2021). In most bivalves, gills have undergone a secondary adaptation to mainly serve as feeding structures while their function in osmoregulation and ion transport have been less studied (Riisgård et al., 2011, 2015; Moreira et al., 2015). Due to their permanent contact with the aquatic environment, the gills of fish and mollusks represent a crucial interface between the aquatic organism and the environment. As such, gills are typically equipped with selective (membrane) barriers that control nutrient uptake and toxic xenobiotic or metabolite elimination (Maetz and García Romeu, 1964; Erickson et al., 2006; Luckenbach and Epel, 2008; Hwang et al., 2011; Armitage et al., 2013; Wang and Wang, 2015). In fish, these barriers are present along the whole digestive system to provide precise control over small molecule uptake. While the digestive anatomy across terrestrial and aquatic vertebrates is highly similar, including a digestive tract with a basic segmentation into esophagus, stomach, midgut, and hindgut, the relative lengths and volumes of each section can differ according to diet and means of nutrient extraction and uptake (Chapman, 1997; Collinder et al., 2009; Karasov and Douglas, 2013; Furness et al., 2015).

In fish and mammals, most nutrients as well as water and ions in the diet are typically absorbed through the epithelial cells of the small and large intestines (Sundell and Rønnestad, 2011; Kiela and Ghishan, 2016). Thereby, macro- and micronutrient uptake is often mediated by simple diffusion, facilitated diffusion, or secondary active transport coupled to an electrochemical gradient (Sundell and Rønnestad, 2011). Conversely, the uptake of dietary toxins, toxicants and other harmful xenobiotics is prevented by a combination of primary active ATP-binding Cassette (ABC) and secondary active solute carrier (SLC) proteins lining the apical and basolateral membrane of the enterocytes (Dietrich et al., 2003; Müller et al., 2017; Nicklisch and Hamdoun, 2020). These membrane proteins effectively regulate xenobiotic bioavailability and often act in concert to either pump compounds into the blood stream for metabolic processing in the liver (primarily SLCs) or to efflux them back into the gut lumen (primarily ABCs) for immediate excretion.

Yet, despite the similarities in intestinal macro- and micro-anatomy between terrestrial and aquatic organisms, little is known about the molecular composition and levels of both nutrient uptake and xenobiotic defense systems along the digestive tract of fish and other aquatic organisms. In addition, there is limited data available on the molecular interactions of aqueous and food-borne contaminants with these transport systems and how these interactions could affect nutrient homeostasis and toxic contaminant bioaccumulation.

## 1. Epithelial Transport in the Digestive System of Aquatic Organisms

One of the key functions of the digestive system is to absorb nutrients from the diet that can be metabolized to provide energy for growth and development. At the same time, the intestinal tract serves as a major environmental barrier to prevent toxic xenobiotic uptake and accumulation. In the gills and intestine of fish and other aquatic organisms, several members of both the solute carrier (SLC) and ATP-Binding Cassette (ABC) family of transporters have been identified to participate in essential physiological functions such as nutrient uptake, ion flux, cell signaling processes, and toxic metabolite and xenobiotic efflux.

### 1.1 Nutrient and Endogenous Substrate Transporters

Transepithelial transport of nutrients in the gastrointestinal tract is typically mediated by secondary active transporters of the solute carrier (SLC) family. In humans, SLCs represent the largest group of secondary active membrane transporters (Hediger et al., 2004, 2013; Almén et al., 2009; Höglund et al., 2011). SLCs are capable of facilitating bidirectional transport (Kottra and Daniel, 2001; Kottra et al., 2002; Winter et al., 2011), though are primarily implicated in cellular uptake of nutrients and ions (Planchamp et al., 2007; Colas et al., 2016; Zhang et al., 2018; Felmlee et al., 2020; Song et al., 2020). Despite their important role in nutrient uptake and metabolic homeostasis, SLC transporters are notoriously understudied in humans and aquatic organisms (Verri et al., 2012; Schlessinger et al., 2013; César-Razquin et al., 2015; Barat et al., 2016).

In fish, SLCs play an important role in organismal development and transport of endogenous compounds to allow for proper development of swim bladders (Kim et al., 2020), inner ears (Liu et al., 2015), kidneys (Schoels et al., 2021), and normal development of embryos (Takesono et al., 2012). In addition, SLCs play a crucial role in intestinal absorption and transport of amino acids and other physiological compounds, including thyroid hormones (Arjona et al., 2011; Muzzio et al., 2014), iron (Cooper et al., 2007), zinc (Yan et al., 2012; Jiang et al., 2014), coenzyme-A (Kim et al., 2020), and amino acids and peptides (Romano et al., 2006, 2014; Verri et al., 2010, 2017; Margheritis et al., 2013; Rimoldi et al., 2015; Song et al., 2017; To et al., 2019; Vacca et al., 2019). Several SLC transporters have been shown to be involved in intestinal methionine absorption as well as iron transport and acid-base regulation in the gills (Cooper et al., 2007; Ivanis et al., 2008; To et al., 2019). For instance, in the intestine of rainbow trout, 8 SLC transporters (SLC1A5, SLC6A19, SLC6A15, SLC7A7, SLC43A1, SLC43A2, SLC3A1, and SLC3A2), which represent both high and low affinity methionine transporters, were found to have differential expression both along the length of the intestine and between the apical and basolateral membranes of the intestine (To et al., 2019). In the gills, sodium/hydrogen exchanging proteins SLC9A2 and SLC9A3, which are highly expressed in the gills and kidney, were found to localize to mitochondria rich cells that were also enriched with sodium/potassium ATPase, indirectly suggesting their involvement in acid-base regulation in rainbow trout (*Oncorhynchus mykiss*) gills (Ivanis et al., 2008). Furthermore, peptide transporting proteins of the SLC15 protein family are highly expressed in zebrafish (*Danio rerio*) intestines (Romano et al., 2006; Vacca et al., 2019). High intestinal expression was also shown for SLC15 proteins in tilapia (*Oreochromis niloticus*) (Huang et al., 2015). In Zebrafish, the expression of SLC22 proteins is low in the intestine, gills and heart while high in the liver and kidney (Mihaljevic et al., 2016). However, Organic Anion Transporting Proteins (OATPs), a subfamily of SLC22 proteins, are highly expressed in the gills and intestine (Popovic et al., 2010a). Intestinal SLC type transporters have also been described to transport vitamins, including vitamin E (alpha-tocopherol), vitamin B1 (thiamine), vitamin C (ascorbic acid), vitamin B7 (biotin), vitamin B9 (folate), and vitamin B2 (riboflavin) (Said and Nexo, 2018). Deficiencies in some of those vitamins have been shown to cause severe organ damage, oxidative stress, and developmental toxicity in fish (Wang et al., 2016; Pan et al., 2017; Harder et al., 2018).

### 1.2 Multidrug/Multixenobiotic Resistance Transporters

Members of both the ABC and SLC-type families have been shown to be part of a highly conserved defense mechanism against chemical insults, often referred to as multidrug-resistance (MDR) transporters (Ling, 1997; Gottesman et al., 2002; Glavinas et al., 2004; Leslie et al., 2005; Sharom, 2008; Giacomini et al., 2010; Hillgren et al., 2013; Nigam, 2015). The mammalian homologs of these transporter superfamilies have been extensively studied in the past, unraveling their precise molecular interactions with drugs and environmental chemicals (Juliano and Ling, 1976; Beck, 1987; Horio et al., 1988; Hediger et al., 2004; Oosterhuis et al., 2008; Staud et al., 2013; Nicklisch et al., 2016; Chedik et al., 2019; Guéniche et al., 2020; Nicklisch and Hamdoun, 2020).

In the early 90s, a mechanism similar to MDR was reported in aquatic organisms and since then referred to as multixenobiotic-resistance or MXR (Kurelec, 1992, 1997; Bard, 2000; Epel et al., 2008; Luckenbach and Epel, 2008; Ferreira et al., 2014). The MXR phenotype and its inducibility by environmental chemicals was first demonstrated in invertebrate species in the 1990s (Cornwall et al., 1995; Kurelec et al., 1995; Smital and Kurelec, 1998). Thereby, baseline levels of MXR inducibility and activity between different species have been correlated with the amount of pollution present in their habitat (Kurelec et al., 1995; Smital et al., 2000). Over the past three decades, fish and other aquatic invertebrates, such as sea urchins and mussels, have been instrumental in understanding MXR transporter interactions with xenobiotics and the chemosensitization effects of so called Transporter-Interfering Chemicals or TICs (Eufemia et al., 2002; Hamdoun et al., 2004; Luckenbach and Epel, 2005, 2008; Kwong et al., 2006; Stevenson et al., 2006; Faria et al., 2011; Martins et al., 2020; Nicklisch and Hamdoun, 2020; Protopopova et al., 2020; Nicklisch et al., 2021).

#### 1.2.1 ABC-Type MDR/MXR Transporters

The ABC-type transporter P-glycoprotein (aka MDR1 or ABCB1) is one the best characterized mammalian MDR/MXR transporters that shows high expression in biological barriers such as the liver, brain, kidney and the intestine (Juliano and Ling, 1976; Beck, 1987; Horio et al., 1988). The identification of two P-glycoprotein genes in the winter flounder *Pleuronectes americanus* using Southern blot techniques represented the first set of P-glycoprotein orthologs reported in lower vertebrates (Chan et al., 1992; Luckenbach et al., 2014; Gordon et al., 2019). Since then, numerous ABC transporter proteins, which mediate the MXR phenotype, have been identified, characterized, and cloned in other fish species including Emerald rockcod (Zucchi et al., 2010), carp (Smital and Sauerborn, 2002), catfish (Liu et al., 2013), killifish (Christine Paetzold et al., 2009), mullet (Diaz de Cerio et al., 2012), Nile tilapia (Costa et al., 2012, 2013), rainbow trout (Lončar et al., 2010; Kropf et al., 2016; Love et al., 2021), zebrafish (Popovic et al., 2010b; Long et al., 2011; Fischer et al., 2013), and more recently yellowfin tuna (Nicklisch et al., 2021).

The mammalian MDR/MXR transporters include the ABCB, ABCC and ABCG subfamilies. Each of these subfamilies is comprised of members with physiological efflux function, xenobiotic efflux function, or both (Dean et al., 2001; Giacomini et al., 2010; König et al., 2013; Nicklisch and Hamdoun, 2020). For instance, in the human ABCB subfamily, ABCB1 has been characterized as a key drug efflux transporter in multiple barrier tissues while ABCB4 mainly serves as phosphatidylcholine transporter in the liver (Borst and Elferink, 2002; Dean and Annilo, 2005; Jonker et al., 2009). The human *abcb5* gene has been suggested to confer drug resistance in malignant melanoma cells (Chen et al., 2005; Frank et al., 2005). Based on synteny analysis, the human *abb1* and *abcb4* genes are co-orthologs of zebrafish *abcb4* (Ferreira et al., 2014; Luckenbach et al., 2014). Notably, several teleost fish, including zebrafish, lack the *abcb1* ortholog but possess the two P-glycoproteins ABCB4 and ABCB5 (Fischer et al., 2013; Luckenbach et al., 2014). Thereby, the Zebrafish ABCB4 transporter has been described as major multixenobiotic efflux transporter while ABCB5 might be implicated in xenobiotic efflux in gill and skin ionocytes (Dymowska et al., 2012; Gordon et al., 2019). Yet, other fish species have been shown to possess *abcb1* and *abcb4* but lack the *abcb5* ortholog (Liu et al., 2013). The confusion in annotating human versus fish MDR/MXR transporter genes mainly arises from lineage-specific genes, i.e., genes that have no detectable homologs in the other species (Dean and Annilo, 2005; Annilo et al., 2006; Gabaldón and Koonin, 2013). For the sake of clarity, in this perspective, we will refer to the aquatic species’ transporter genes as they have been annotated by the cited authors. Future combined synteny and phylogenetic analysis will likely revise those annotations and provide means to reduce the difficulty of inferring transporter function and nomenclature.

Studies on localization and expression levels of fish ABC-type MDR/MXR transporters have mostly focused on two model organisms, rainbow trout and zebrafish. For instance, the qPCR analysis of eight different ABC transporters (*abcb1, abcb11, abcc1, abcc2, abcc3, abcc4, abcc5*, and *abcg2*) in the liver, brain, gonads, kidney, gills, and intestine of rainbow trout revealed similar expression patterns to mammalian tissues (Lončar et al., 2010). Thereby, relative expression was higher in digestive and excretory tissues such as the liver, kidneys, and intestine compared to the brain or gills. Interestingly, the expression of the key efflux pump *abcb1* was highest in brain but not detected in the gills (Lončar et al., 2010). The bile salt pump *Abcb11* had very low expression across the tested tissues except for the liver.

The mRNA levels of *abcb1a, abcb1b, abcc1, abcc2, abcc3, abcc4, abcc5, and abcg2* were also examined in earlier stages of rainbow trout embryo development and increased from 1 to 20 days post hatch. The predominant increases in *abcb1a* and *abcb1b* gene expression occurred in abdominal viscera (intestine, liver, kidney) and head (gills) of early larvae (Kropf et al., 2016). Gills of early larvae were also high in *abcb5*, *abcc3*, and *abcg2* mRNA (Kropf et al., 2020). The ABC proteins *abcb4, abcb5, abcc2* and *abch1* have also been characterized in zebrafish and have been shown to confer the MXR phenotype (Popovic et al., 2010b; Long et al., 2011; Fischer et al., 2013). Cellular ABC transporter efflux activities have been detected in zebrafish embryos within 48 h of development (Long et al., 2011; Fischer et al., 2013). The *abch1* transporter gene is unique to vertebrate fish and showed higher expression in tissues such as the gills and brain than in the intestine or liver (Popovic et al., 2010b). Expression of the *abcc2* gene was highest in the intestine, followed by the kidney and liver (Long et al., 2011).

#### 1.2.2 SLC-Type MDR/MXR Transporters

The SLC proteins are responsible for transporting a wide range of substrates including both organic and metal ions, endogenous metabolites, and xenobiotics such as pharmaceuticals and toxicants (Hediger et al., 2004; König et al., 2013; Lin et al., 2015; Bruyere et al., 2017; Chedik et al., 2019; Nicklisch and Hamdoun, 2020; Pizzagalli et al., 2021). In clinical research, there has been particular focus on members of the SLC22 family, which encompasses many organic ion transporters, and the SLC47 family, the multidrug and toxicant extrusion (MATE) proteins (Nigam, 2015, 2018). Yet, similar to nutrient uptake transporters, there have been very few studies on the tissue expression levels and localization of MDR/MXR-type SLC transporters in aquatic organisms. In the earlier stages of rainbow trout embryo development, the multidrug and toxin extrusion protein 1 (*mate1)* and organic anion transporting polypeptide 2 (*oatp2*) genes were detected in yolk sac epithelium, while the mRNA levels of the organic anion transporting polypeptide 1d gene (*oatp1d*) were high in abdominal viscera (Kropf et al., 2016). During the development of zebrafish embryos, *mate6* and *mate7* genes are highly expressed after 6 hpf (Lončar et al., 2016). Interestingly, tissue-specific *mate* gene expression in kidney, liver, intestine, and brain was generally higher in male than in female zebrafish, indicating gender differences in these important detoxification systems. The lowest gene expression of *mate3, 4, 5, 6, 7, and 8* was found in the gills, irrespective of sex.

## 2. Regulation of Nutrient and MDR/MXR Transporter Expression

### 2.1 Diet and Adaptive Regulation of Intestinal Uptake

The quantity and quality of diet have been shown to influence transporter expression levels in the intestine of fish. For instance, fasting of Nile tilapia (*Oreochromis niloticus*) was shown to significantly decrease mRNA levels of the SLC15 peptide transporter gene family, but mRNA levels recovered upon refeeding (Huang et al., 2015). Similar results were seen in the Mummichog (*Fundulus heteroclitus*), where short-term fasting increased, long-term fasting decreased, and re-feeding restored SLC15 mRNA levels (Bucking and Schulte, 2012). Further, short-term starvation of rainbow trout induced an increase of P-glycoprotein concentration in the intestinal epithelia (Baumgarner et al., 2013).

Diet quality has also been shown to have a pronounced effect on fish transporters, and is particularly relevant in the field of aquaculture, where farmers have direct control over the primary source of nutrition as well as any form of dietary supplementation. Replacement of traditional fishmeal with meat and bone meal in the diet of juvenile turbot (*Scophthalmus maximus*) was shown to increase intestinal peptide transporter 1 (PepT1/SLC15A1) (Song et al., 2017). Similarly, large reductions in fishmeal content and replacement with vegetable meal in European sea bass (*Dicentrarchus labrax*) diet was shown to increase PepT1 gene expression but not affect the expression of the monocarboxylate transporter SLC6A19 (Rimoldi et al., 2015). Atlantic salmon (*Salmo salar*) fed diets supplemented with saponins and pea protein were shown to have several metabolic systems greatly altered, including differential expression of several SLC proteins (Kortner et al., 2012). Studies on dietary restrictions and feed supplementation that may decrease fitness of farmed fish are still in the early stages, but could serve as guideline to help elucidate mechanisms leading to various forms of intestinal pathology (Kortner et al., 2012).

### 2.2 Environmental Stressors

As a result of climate change and associated alterations in salinity, pH, and temperature of aquatic environments, the bioavailability and toxicity of environmental chemicals can increase (Noyes et al., 2009; Hasenbein et al., 2018; Ross and Behringer, 2019; Paul et al., 2020; Derby et al., 2021; Fulton et al., 2021). Recent studies have shown that these environmental stressor can also affect gene regulation, expression, and overall fitness of fish populations both within and between species (Jeffries et al., 2018, 2019; DeCourten et al., 2019; Romney et al., 2019; Mundy et al., 2020; Komoroske et al., 2021; Segarra et al., 2021). Most of these investigations have been conducted in fish and focused on SLC-type transporters and the effect of pH and temperature on substrate affinity and transport rate.

For instance, increased pH (pH > 7.4) was shown to cause a decrease in copper absorption in rainbow trout gut sacs as well as a decrease in iron absorption by SLC11 proteins isolated from rainbow trout gills and expressed in *Xenopus laevis* oocytes (Cooper et al., 2007; Nadella et al., 2007). The pH dependence of PepT1 in teleost fish has been thoroughly examined. In Zebrafish and other teleost fish, PepT1 tends to exhibit a higher substrate affinity with decreasing pH (Romano et al., 2014; Verri et al., 2017). Zebrafish PepT1 also exhibited an increase in maximal transport rate when extracellular pH transitioned from acidic (pH = 6.5) to alkaline (pH = 8.5), while other teleost fish Pept1 proteins exhibit little or no change in maximal transport rate in response to similar pH shifts (Romano et al., 2014; Verri et al., 2017).

The transport rate of SLC transporters at different temperatures has been shown to vary by species (Romano et al., 2014; Verri et al., 2017). Interestingly, teleost fish PepT1 proteins have higher transport rates at lower temperatures (22°C) than the mammalian PepT1 orthologs, which function better at temperatures around 30°C and higher. This possibly demonstrates an adaptative evolution between terrestrial and aquatic species living in different temperature regimens (Romano et al., 2014; Verri et al., 2017). The Antarctic icefish (*Chionodraco hamatus*) represents an extreme example, with structural changes to its PEPT1 protein facilitating in part its ability to function at very cold (−1.9°C) temperatures (Romano et al., 2014; Verri et al., 2017). In Delta smelt (*Hypomesus transpacificus*), the expression of the ion exchange transporter SLC8B1 was shown to have non-linear responses to increasing temperature, with decreased expression at 20°C and 25°C compared to the control (14°C) and re-elevated expression at 27°C (Jeffries et al., 2018). This u-shaped response was attributed to sublethal thermal threshold with partial downregulation of non-essential cellular processes during the period of stress.

If and to what extent ABC-type MDR/MXR transporters can be regulated by changes in salinity, temperature or pH is not well understood. For mammalian P-glycoprotein (ABCB1), a linear increase in transport rate with increasing temperature has been demonstrated (Litman et al., 1997; Lu et al., 2001; Clay and Sharom, 2013). The authors suggested that the increase in drug transport rate at higher temperatures can be attributed to several factors, including the increased partitioning of the drug/xenobiotic into the membrane and the structural changes in the protein that increase substrate affinity. Effects of other environmental stressors on the affinity and/or transport rate of ABC transporters are still elusive.

### 2.3 Chemical Inducers and Repressors

Multixenobiotic Resistance transporters represent a critical line of defense in preventing xenobiotic chemicals from accumulating and harming an aquatic organism. Initial investigations identifying MXR transporters as molecular targets of xenobiotics focused on chemical exposure experiments and subsequent evaluation of transporter mRNA and/or protein expression levels (Table 1). Thereby, transporter downregulation suggested a mechanism of direct inhibition or that upstream or downstream regulatory pathways might be targeted by the compound. Whereas upregulation of transporters indicated that they could be involved in the elimination of those compounds.

**TABLE 1:** List of tissue specific MXR transporter gene induction or repression by environmental chemicals, heavy metals, and natural toxins.

Compounds	Transporter(s)	Genetic effects	Organism	Tissue	References
Cadmium chloride (CdCl_2_)	ABCB1, ABCC2	Induction	Emerald rockcod (*Trematomus bernacchii*)	Liver	Zucchi et al., 2010
Mercury (Hg)	ABCC2	Induction	Zebrafish (*Danio rerio*)	Liver, kidney, intestine	Long et al., 2011
Lead (Pb)
Mercury chloride (HgCl_2_)	ABCG2b	Induction	Zebrafish (*Danio rerio*)	Intestine	Zhang et al., 2020
Gossypol	SLC6A6, SLC1A2a, SLC1A3, SLC7A7, SLC7A6, SLC7A1, SLC6A19b, SLC7A5, SLC7A8, SLC1A5, SLC38A2, PepT1	Repression	Grass carp (*Ctenopharyngodon idella*)	Intestine	Wang et al., 2018
Clotrimazole (CTZ)	ABCB1b	Induction	Rainbow trout (*Oncorhynchus mykiss*)	Optic lobe, distal intestine	Love et al., 2021
Copper (Cu^2 +^)	SLC15a1b	Induction	Nile tilapia (*Oreochromis niloticus*)	Proximal intestine	Huang et al., 2015
Mercury (Hg^2 +^)
Cadmium telluride quantum dots (CdTe-QDs)	MRP1, MRP2,	Induction	Zebrafish (*Danio rerio*)	Whole embryo	Tian et al., 2019;
Cadmium (Cd^2 +^)	P-glycoprotein	Hu et al., 2019
Silver (Ag^+^)		
Pentachlorophenol (PCP)	ABCB1	Induction	Water flea (*Daphnia magna)*	Whole organism	Campos et al., 2014
Mercury chloride (HgCl_2_)	ABCC4	Induction			
Okadaic acid (OA)	P-glycoprotein (ABCB11),	Induction	Mediterranian mussel (*Mytilus*	Digestive glands, gills	Martins et al., 2020;
Dinophysistoxin-1 (DTX1)	SLC6A7, SFXN1, MDR1, MRP2	*galloprovincialis*)		Martínez-Escauriaza et al., 2021
Benzo(α)pyrene (BaP)	ABCB1, ABCC	Induction	Korean mussel (*Mytilus coruscus*)	Gills	Guo et al., 2020

In rainbow trout, the expression of two P-glycoprotein isoforms, *abcb1a* and *abcb1b*, was significantly increased upon exposure to the antifungal agent clotrimazole (Love et al., 2021). In addition, several heavy metals were shown to induce the expression of ABC proteins and act as their substrates in fish. For instance, both cadmium and silver ions have been shown to induce *pgp* (ABCB4) and *abcc2* expression in zebrafish embryos (Hu et al., 2019). Mercury and lead induced *abcc2* expression in both larval and adult zebrafish, and the overexpression of *abcc2* alleviated the accumulation of these metals along with cadmium (Long et al., 2011). In the Antarctic fish species Emerald rockcod (*Trematomus bernacchii*), the *abcb1* and *abcc2* genes are induced in the liver upon exposure to cadmium ions (Zucchi et al., 2010). Cadmium telluride quantum dots (CdTe-QDs) are nanoparticles that are often released into environmental water bodies and accumulate in aquatic biota. Zebrafish embryos exposed to CdTe quantum dots show delayed hatching and induce the expression of *abcc1* and *abcc2*, indicating that quantum dots could be possible substrates for these ABCC-type transporters (Tian et al., 2019).

There has been limited studies on the inducing or repressing effects of xenobiotics on SLC transporter expression. In Nile tilapia (*Oreochromis niloticus*) the expression levels of peptide uptake transporters SLC15a1a, SLC15a1b, SLC15a2, and SLC15a5 decreased after fasting (Huang et al., 2015). Subsequent exposure to waterborne copper, but not mercury, prevented the restoration of the expression levels. In young grass carp (*Ctenopharyngodon idella*), the cotton derived phenol gossypol was shown to down-regulate several peptide and amino acid SLC transporters in the intestine, leading to intestinal damage and reduced growth (Wang et al., 2018). This is particularly important for aquaculture species where commercially available plant protein sources like rapeseed and cottonseed meal are used as cost effective feed (Deng et al., 2014). Unintentional exposure to gossypol in cottonseed meal could ultimately reduce feeding efficiency and overall percent weight gain.

## 3. Molecular Interactions of Xenobiotics With MXR Transporters

To date, the number of identified MXR transporter substrates and inhibitors among xeobiotics is low and assay protocols have yet to be standardized (Table 2; Nicklisch and Hamdoun, 2020). Still, several environmental chemicals, including pharmaceuticals, pesticides, herbicides, industrial chemicals, and polycyclic aromatic hydrocarbons (PAHs) have been shown to interact with and inhibit the function of MXR transporters in fish, bivalves, and crustaceans.

**TABLE 2:**
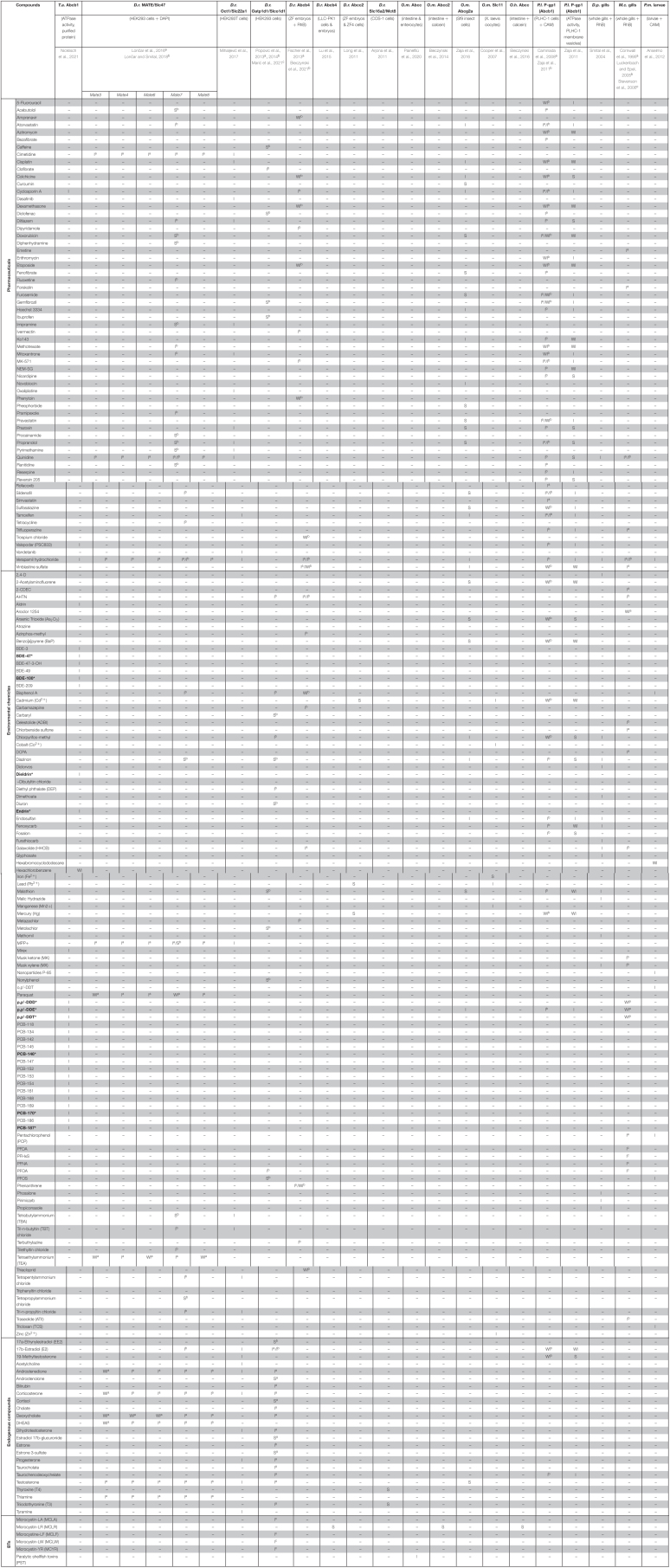
Summary of known xenobiotic interactions with MXR transporters in fish and aquatic invertebrates.

Compounds marked with an asterisk (*) have been previously identified as Transporter-Interfering Chemicals or TICs (Nicklisch et al., 2016, 2021; Nicklisch and Hamdoun, 2020). T.a., Thunnus albacares; D.r., Danio rerio; O.m, Oncorhynchus mykiss; O.h., Odontesthes hatcheri; P.l., Poeciliopsis lucida; D.p., Dreissena polymorpha; M.c., Mytilus californianus; P.m., Psammechinus miliaris; S, substrate; I, inhibitor; WI, weak interactor (i.e., compounds that are not recognized or weakly interact with MXR transporters and do not alter transporter activity or function (Nicklisch and Hamdoun, 2020); BTs, biotoxins; NEM-SG, N-ethylmaleimide S-glutathione; CDEC, chloroallyl diethyldithiocarbamate; AHTN, Acetyl-hexamethyltetrahydro-naphthalene; DCPA, Dimethyl tetrachloroterephthalate; MPP+, 1-methyl-4-phenylpyridinum; 2,4-D, 2,4-dichlorophenoxyacetic acid; DHEAS, dehydroepiandrosterone sulfate; PFDA, Perfluorodecanoic acid; PFHxS, Perfluorohexanesulfonate; PFNA, Perfluorononanoic acid; PFOS, Perfluorooctanesulfonic acid; PFOA, Perfluorooctanoic acid.

For instance, in PLHC-1 (*Poeciliopsis lucida hepatoma cells*) the ABCB1 homolog P-gp1 was shown to be inhibited by a series of pharmaceuticals, with sildenafil and simvastatin showing highest inhibition potency (Caminada et al., 2008). In zebrafish, ABCB4 efflux function was inhibited by different environmental chemicals, including insecticides, fragrances, and pharmaceuticals (Fischer et al., 2013; Bieczynski et al., 2021). Divalent metals (Cd, Mn, Zn, Pb, and Co) have been shown to cause (competitive) inhibition of rainbow trout SLC11 iron uptake in *Xenopus* oocytes (Cooper et al., 2007). Using HEK293 cells, heterologously expressing zebrafish MATE7/SLC47a7 protein, interactions with 89 different environmental chemicals were investigated with the majority of compounds being inhibitors (Lončar and Smital, 2018). Similarly, the model substrate transport of zebrafish organic anion transporting polypeptide 1d1 (OATP1d1) and organic cation transporter 1 (OCT1) expressed in HEK293 cells was shown to be (competitively) inhibited by nearly all tested endo- and xenobiotics (Popovic et al., 2014; Mihaljević et al., 2017).

In bivalves, exposures to organic pesticides, pharmaceuticals, industrial chemicals, cadmium, and benzo(a)pyrene led to increased deformities, impaired growth, and increased toxicant accumulation. For instance, in the Californian mussel (*Mytilus californianus*), moderately hydrophobic pesticides and PFAS compounds have been shown to inhibit gill tissue efflux of Rhodamine B, a known P-glycoprotein substrate (Cornwall et al., 1995; McFadzen et al., 2000; Stevenson et al., 2006). In the thick shell mussel (*Mytilus coruscus*), gill tissue pre-exposure to benzo(α)pyrene (BaP) reduced the inhibitory effect of ABCB1- and ABCC1-specific inhibitors reversin 205 and MK572 on Calcein-AM efflux (Guo et al., 2020). This indicated that BaP could bind to both transporters and possible alter their detoxification function in these mussels.

Using mussels, sea urchin larvae, and the water flea *Daphnia magna*, it was shown that MXR transporter inhibition may also facilitate toxic synergism in environmental chemical mixtures (Faria et al., 2011; Anselmo et al., 2012; Campos et al., 2014). For instance, when early life stages of the green sea urchin (*Psammechinus miliaris*) were exposed to combinations of the toxic MXR transporter substrates vinblastine and triclosan (TCS) or P-85 nanoparticles, the toxicity increased by a factor of 2-8 (Anselmo et al., 2012). Similarly, when early life stages of the zebra mussel (*Dreissena polymorpha*) were exposed to a combination of vinblastine and model ABC transporter inhibitors, the toxicity increased super-additively (Faria et al., 2011).

Further, marine biotoxins and other naturally occurring marine products have been shown to upregulate ABC and SLC protein expression and function as substrates in various mussels species (Eufemia et al., 2002; Martins et al., 2020). *In vitro* and *ex vivo* experiments in rainbow trout and the Patagonian silverside (*Odontesthes hatcheri*) suggest that microcystin-LR may act as a substrate and competitive inhibitor of ABCC-like proteins in intestinal tissues (Bieczynski et al., 2014, 2016). Additional *ex vivo* and *in vitro* experiments in rainbow trout intestines demonstrated that paralytic shellfish toxin (PST) is capable of inhibiting ABCC-like transporters when absorbed by intestinal epithelial cells (Painefilú et al., 2020). The activity of ABC proteins can also be regulated by endogenous compounds. For instance, in the rectal salt glands of dogfish sharks (*Squalus acanthias*), ABCC2 activity was shown to be inhibited by the vasoconstrictor endothelin-1 via the ET_*B*_ receptor and protein kinase C signaling (Miller et al., 2002).

## 4. Implications of Transporter Inhibition for Chemical Bioaccumulation and Nutrient Deficiencies

Many of the environmental chemicals that have been tested with MDR/MXR and nutrient uptake transporters were shown to be competitive substrates or inhibitors of the transporters in aquatic organisms. These so-called Transporter-Interfering Chemicals or TICs had been previously identified and shown to bind and inhibit mammalian and fish defense transporters, such as ABCB1 (Nicklisch et al., 2016, 2021; Nicklisch and Hamdoun, 2020). It has been hypothesized that the exact type of transporters also serve endogenous roles during development and for maintaining cellular and organismal homeostasis (Ahn and Nigam, 2009; Wu et al., 2011; Nigam, 2015, 2018). In addition, the known substrate overlaps and structural similarities between serval nutrient and MDR/MXR transporter subfamilies would likely make nutrient uptake systems another target for TICs (Kaler et al., 2007; Truong et al., 2008; Ahn and Nigam, 2009). This is critical since the inhibition of both xenobiotic defense systems and nutrient uptake transporters can potentiate detrimental effects on a developing aquatic organism. Nutrient deficient animals, specifically with vitamin deficiencies, have been shown to grow slower and suffer from other conditions such as hypoxia, neurological disorders, immunosuppression, and lower reproductive viability (Deng et al., 2014; Wang et al., 2016; Pan et al., 2017; Harder et al., 2018; Talukder Shefat, 2018). Likewise, the inhibition of xenobiotic efflux transporters will further promote the accumulation of toxic substrates that are otherwise eliminated.

Hence, contaminant-laden diets can represent a critical exposure pathway for fish to toxic environmental chemicals and natural compounds (Streit, 1998; Mackay and Fraser, 2000; Macdonald et al., 2002; Armitage et al., 2013; Mackay et al., 2018). This is particularly relevant in aquaculture, as it has been demonstrated that farmed fish can carry higher pollutant loads than wild caught fish (Hites et al., 2004a,b). Feed contamination is a prevalent issue and includes persistent organic pollutants, such as polybrominated diphenyl ethers (PBDEs) and polychlorinated biphenyls (PCBs), and heavy metals such as mercury, arsenic, cadmium, iron, and lead (Choi and Cech, 1998; Dórea, 2006; Kundu et al., 2017; Biancarosa et al., 2019; Li et al., 2019). While individual levels of these contaminants in fish feed or aquatic environments might be low, local tissue concentration in the digestive tract of these mostly hydrophobic compounds and the synergistic action of their mixtures can effectively act in concert to inhibit the protective efflux function of intestinal transport systems.

As such, it is important to both expand the panel of tested environmental chemicals and to include physiological transporters to evaluate the impacts on nutrient uptake, metabolism, and homeostasis. Furthermore, the transporter/chemical interaction assays need to be standardized and include co-exposures to chemical mixtures, representing environmental levels found in fish feed and/or the aquatic environment. Finally, given the differences in MDR/MXR transporter sequences between model and commercial fish species (Nicklisch et al., 2021), it is important to include MXR and nutrient transporters from commercial fish species to better predict and mitigate toxic chemical and heavy metal bioaccumulation fish and seafood species (Nicklisch et al., 2017a,b). Together, such studies will inform fish and shellfish advisories on guidelines for the selection of TIC-free aquaculture feed to develop safe eating guidelines.

## Conclusion and Future Directions

To date only a handful of MDR/MXR transporters have been cloned and functionally characterized in fish and aquatic invertebrates. Among those, the least characterized MXR transporter superfamily are the solute carriers (SLCs). Transporter localization, gene expression levels and endogenous and xenobiotic substrate/inhibitor identification has mainly focused on two model organisms: zebrafish and rainbow trout. Furthermore, there have been only a few studies conducted on fish feed and dietary levels of contaminants and transporter-mediated uptake and bioaccumulation in commercial fish and other human-relevant aquaculture species. Finally, the extent to which other mutable environmental factors, such as salinity, temperature, and pH, may impair the levels and function of MXR and other nutrient transporters remains elusive. This perspective further highlights the urgent need to identify novel TICs, to determine their environmental levels in the natural diets and feeds of fish and other aquatic invertebrates, and to specify the transporter targets that regulate their uptake and disposition in aquatic organisms. Special emphasis should be placed on determining the interactions of TICs with protective MXR efflux transporters and essential nutrient uptake transporters lining the gills and intestinal epithelia to better predict toxic chemical accumulation and/or nutrient deprivation. The key strategies to mitigate toxicant and toxin accumulation in aquatic organisms include targeted chemical analysis of fish feed in aquaculture operations, a continuous biomonitoring of TIC levels in lower trophic level organisms that serve as prey or feed for commercial fish species and the development of regulatory guidelines that inform industrial chemical and agricultural pesticide management strategies to reduce or eliminate the use of environmentally persistent TIC compounds.

## Data Availability Statement

The original contributions presented in the study are included in the article/supplementary material, further inquiries can be directed to the corresponding author/s.

## Author Contributions

Both authors listed have made a substantial, direct, and intellectual contribution to the work, and approved it for publication.

## Conflict of Interest

The authors declare that the research was conducted in the absence of any commercial or financial relationships that could be construed as a potential conflict of interest.

## Publisher’s Note

All claims expressed in this article are solely those of the authors and do not necessarily represent those of their affiliated organizations, or those of the publisher, the editors and the reviewers. Any product that may be evaluated in this article, or claim that may be made by its manufacturer, is not guaranteed or endorsed by the publisher.

## References

[B1] Ahn S. Y. Nigam S. K. (2009). Toward a systems level understanding of organic anion and other multispecific drug transporters: a remote sensing and signaling hypothesis. *Mol. Pharmacol.* 76 481–490. 19515966 10.1124/mol.109.056564 PMC2730381

[B2] Almén M. S. Nordström K. J. Fredriksson R. Schiöth H. B. (2009). Mapping the human membrane proteome: a majority of the human membrane proteins can be classified according to function and evolutionary origin. *BMC Biol.* 7:50. 10.1186/1741-7007-7-50 19678920 PMC2739160

[B3] Annilo T. Chen Z.-Q. Shulenin S. Costantino J. Thomas L. Lou H. (2006). Evolution of the vertebrate ABC gene family: analysis of gene birth and death. *Genomics* 88 1–11. 10.1016/j.ygeno.2006.03.001 16631343

[B4] Anselmo H. M. R. van den Berg J. H. J. Rietjens I. M. C. M. Murk A. J. (2012). Inhibition of cellular efflux pumps involved in multi xenobiotic resistance (MXR) in echinoid larvae as a possible mode of action for increased ecotoxicological risk of mixtures. *Ecotoxicology* 21 2276–2287. 10.1007/s10646-012-0984-2 22868905

[B5] Arjona F. J. de Vrieze E. Visser T. J. Flik G. Klaren P. H. M. (2011). Identification and functional characterization of zebrafish solute carrier Slc16a2 (Mct8) as a thyroid hormone membrane transporter. *Endocrinology* 152 5065–5073. 10.1210/en.2011-1166 21952246

[B6] Armitage J. M. Arnot J. A. Wania F. Mackay D. (2013). Development and evaluation of a mechanistic bioconcentration model for ionogenic organic chemicals in fish. *Environ. Toxicol. Chem.* 32 115–128. 10.1002/etc.2020 23023933

[B7] Barat A. Sahoo P. K. Kumar R. Pande V. (2016). Solute carriers (SLCs) identified and characterized from kidney transcriptome of golden mahseer (Tor putitora) (Fam: cyprinidae). *Comparat. Biochem. Physiol. Part B: Biochem. Mol. Biol.* 200 54–61. 10.1016/j.cbpb.2016.06.003 27287540

[B8] Bard S. M. (2000). Multixenobiotic resistance as a cellular defense mechanism in aquatic organisms. *Aquatic Toxicol.* 48 357–389. 10.1016/S0166-445X(00)00088-6 10794825

[B9] Baumgarner B. L. Bharadwaj A. S. Inerowicz D. Goodman A. S. Brown P. B. (2013). Proteomic analysis of rainbow trout (Oncorhynchus mykiss) intestinal epithelia: physiological acclimation to short-term starvation. *Comparat. Biochem. Physiol. Part D, Genomics Proteomics* 8 58–64. 10.1016/j.cbd.2012.11.001 23261852

[B10] Beck W. T. (1987). The cell biology of multiple drug resistance. *Biochem. Pharmacol.* 36 2879–2887. 10.1016/0006-2952(87)90198-5 2888464

[B11] Biancarosa I. Sele V. Belghit I. Ørnsrud R. Lock E.-J. Amlund H. (2019). Replacing fish meal with insect meal in the diet of Atlantic salmon (Salmo salar) does not impact the amount of contaminants in the feed and it lowers accumulation of arsenic in the fillet. *Food Additives Contaminants: Part A* 36 1191–1205. 10.1080/19440049.2019.1619938 31161892

[B12] Bieczynski F. Burkhardt-Medicke K. Luquet C. M. Scholz S. Luckenbach T. (2021). Chemical effects on dye efflux activity in live zebrafish embryos and on zebrafish Abcb4 ATPase activity. *FEBS Lett.* 595 828–843. 10.1002/1873-3468.14015 33274443

[B13] Bieczynski F. De Anna J. S. Pirez M. Brena B. M. Villanueva S. S. M. Luquet C. M. (2014). Cellular transport of microcystin-LR in rainbow trout (Oncorhynchus mykiss) across the intestinal wall: possible involvement of multidrug resistance-associated proteins. *Aquatic Toxicol. (Amsterdam, Netherlands)* 154 97–106. 10.1016/j.aquatox.2014.05.003 24865614

[B14] Bieczynski F. Torres W. D. C. Painefilu J. C. Castro J. M. Bianchi V. A. Frontera J. L. (2016). Alterations in the intestine of Patagonian silverside (Odontesthes hatcheri) exposed to microcystin-LR: changes in the glycosylation pattern of the intestinal wall and inhibition of multidrug resistance proteins efflux activity. *Aquatic Toxicol.* 178 106–117. 10.1016/j.aquatox.2016.07.016 27474942

[B15] Borst P. Elferink R. O. (2002). Mammalian ABC transporters in health and disease. *Annu. Rev. Biochem.* 71 537–592. 10.1146/annurev.biochem.71.102301.093055 12045106

[B16] Bruyere A. Hubert C. Le Vee M. Chedik L. Sayyed K. Stieger B. (2017). Inhibition of SLC drug transporter activities by environmental bisphenols. *Toxicol. Vitro: Int. J. Published Assoc. ith BIBRA* 40 34–44. 10.1016/j.tiv.2016.12.009 27989701

[B17] Bucking C. Schulte P. M. (2012). Environmental and nutritional regulation of expression and function of two peptide transporter (PepT1) isoforms in a euryhaline teleost. *Comparat. Biochem. Physiol. Part A: Mol. Integrat. Physiol.* 161 379–387. 10.1016/j.cbpa.2011.12.008 22227314

[B18] Caminada D. Zaja R. Smital T. Fent K. (2008). Human pharmaceuticals modulate P-gp1 (ABCB1) transport activity in the fish cell line PLHC-1. *Aquatic Toxicol.* 90 214–222. 10.1016/j.aquatox.2008.08.013 18950875

[B19] Campos B. Altenburger R. Gómez C. Lacorte S. Piña B. Barata C. (2014). First evidence for toxic defense based on the multixenobiotic resistance (MXR) mechanism in Daphnia magna. *Aquatic Toxicol.* 148 139–151. 10.1016/j.aquatox.2014.01.001 24486881

[B20] Carvalho O. (2011). Comparative physiology of the respiratory system in the animal kingdom. *Open Biol. J.* 4 35–46. 10.2174/1874196701104010035

[B21] César-Razquin A. Snijder B. Frappier-Brinton T. Isserlin R. Gyimesi G. Bai X. (2015). A call for systematic research on solute carriers. *Cell* 162 478–487. 10.1016/j.cell.2015.07.022 26232220

[B22] Chan K. M. Davies P. L. Childs S. Veinot L. Ling V. (1992). P-glycoprotein genes in the winter flounder, Pleuronectes americanus: isolation of two types of genomic clones carrying 3’ terminal exons. *Biochim. Biophys. Acta* 1171 65–72. 10.1016/0167-4781(92)90140-u 1358208

[B23] Chapman H. W. (1997). Comparative physiology of the vertebrate digestive system, 2nd ed. *Can. Vet. J.* 38 576–577.

[B24] Chedik L. Bruyere A. Fardel O. (2019). Interactions of organophosphorus pesticides with solute carrier (SLC) drug transporters. *Xenobiotica* 49 363–374. 10.1080/00498254.2018.1442030 29448871

[B25] Chen K. G. Szakács G. Annereau J.-P. Rouzaud F. Liang X.-J. Valencia J. C. (2005). Principal expression of two mRNA isoforms (ABCB 5α and ABCB 5β) of the ATP-binding cassette transporter gene ABCB 5 in melanoma cells and melanocytes. *Pigment Cell Res.* 18 102–112. 10.1111/j.1600-0749.2005.00214.x 15760339 PMC3915408

[B26] Choi M. H. Cech J. J. Jr. (1998). Unexpectedly high mercury level in pelleted commercial fish feed. *Environ. Toxicol. Chem.* 17 1979–1981. 10.1002/etc.5620171013

[B27] Christine Paetzold S. Ross N. W. Richards R. C. Jones M. Hellou J. Bard S. M. (2009). Up-regulation of hepatic ABCC2, ABCG2, CYP1A1 and GST in multixenobiotic-resistant killifish (Fundulus heteroclitus) from the Sydney Tar Ponds, Nova Scotia, Canada. *Mar. Environ. Res.* 68 37–47. 10.1016/j.marenvres.2009.04.002 19443023

[B28] Clay A. T. Sharom F. J. (2013). Lipid bilayer properties control membrane partitioning, binding, and transport of P-Glycoprotein substrates. *Biochemistry* 52 343–354. 10.1021/bi301532c 23268645

[B29] Colas C. Ung P. M.-U. Schlessinger A. (2016). SLC transporters: structure, function, and drug discovery. *MedChemComm (RSC Publishing)* 7 1069–1081. 10.1039/C6MD00005C 27672436 PMC5034948

[B30] Collinder E. Björnhag G. Cardona M. Norin E. Rehbinder C. Midtvedt T. (2009). Gastrointestinal host-microbial interactions in mammals and fish: comparative studies in man, mice, rats, pigs, horses, cows, elk, reindeer, salmon and cod. *Microb. Ecol. Health. Dis.* 15 66–78. 10.1080/08910600310014980

[B31] Cooper C. A. Shayeghi M. Techau M. E. Capdevila D. M. MacKenzie S. Durrant C. (2007). Analysis of the rainbow trout solute carrier 11 family reveals iron import ⩽ pH 7.4 and a functional isoform lacking transmembrane domains 11 and 12. *FEBS Lett.* 581 2599–2604. 10.1016/j.febslet.2007.04.081 17509573

[B32] Cornwall R. Toomey B. H. Bard S. Bacon C. Jarman W. M. Epel D. (1995). Characterization of multixenobiotic/multidrug transport in the gills of the mussel Mytilus californianus and identification of environmental substrates. *Aquatic Toxicol.* 31 277–296. 10.1016/0166-445X(94)00070-7

[B33] Costa J. Reis-Henriques M. A. Castro L. F. C. Ferreira M. (2012). Gene expression analysis of ABC efflux transporters, CYP1A and GSTα in Nile tilapia after exposure to benzo(a)pyrene. *Comparat. Biochem. Physiol. Part C: Toxicol. Pharmacol.* 155 469–482. 10.1016/j.cbpc.2011.12.004 22227637

[B34] Costa J. Reis-Henriques M. A. Wilson J. M. Ferreira M. (2013). P-glycoprotein and CYP1A protein expression patterns in Nile tilapia (Oreochromis niloticus) tissues after waterborne exposure to benzo(a)pyrene (BaP). *Environ. Toxicol. Pharmacol.* 36 611–625. 10.1016/j.etap.2013.05.017 23834963

[B35] Dean M. Annilo T. (2005). Evolution of the ATP-binding cassette (ABC) transporter superfamily in vertebrates. *Annu. Rev. Genomics Hum. Genet.* 6 123–142. 10.1146/annurev.genom.6.080604.162122 16124856

[B36] Dean M. Hamon Y. Chimini G. (2001). The human ATP-binding cassette (ABC) transporter superfamily. *J. Lipid Res.* 42 1007–1017. 10.1016/S0022-2275(20)31588-1 11441126

[B37] DeCourten B. M. Connon R. E. Brander S. M. (2019). Direct and indirect parental exposure to endocrine disruptors and elevated temperature influences gene expression across generations in a euryhaline model fish. *PeerJ* 7:e6156. 10.7717/peerj.6156 30643694 PMC6329337

[B38] Deng J. Zhang X. Long X. Tao L. Wang Z. Niu G. (2014). Effects of dietary cholesterol supplementation on growth and cholesterol metabolism of rainbow trout (Oncorhynchus mykiss) fed diets with cottonseed meal or rapeseed meal. *Fish. Physiol. Biochem.* 40 1827–1838. 10.1007/s10695-014-9971-2 25119853

[B39] Derby A. P. Fuller N. W. Huff Hartz K. E. Segarra A. Connon R. E. Brander S. M. (2021). Trophic transfer, bioaccumulation and transcriptomic effects of permethrin in inland silversides, Menidia beryllina, under future climate scenarios. *Environ. Pollut.* 275:116545. 10.1016/j.envpol.2021.116545 33578317

[B40] Diaz de Cerio O. Bilbao E. Cajaraville M. P. Cancio I. (2012). Regulation of xenobiotic transporter genes in liver and brain of juvenile thicklip grey mullets (Chelon labrosus) after exposure to Prestige-like fuel oil and to perfluorooctane sulfonate. *Gene* 498 50–58. 10.1016/j.gene.2012.01.067 22343007

[B41] Dietrich C. G. Geier A. Oude Elferink R. P. J. (2003). ABC of oral bioavailability: transporters as gatekeepers in the gut. *Gut* 52 1788–1795. 14633964 10.1136/gut.52.12.1788 PMC1773875

[B42] Dórea J. G. (2006). Fish meal in animal feed and human exposure to persistent bioaccumulative and toxic substances. *J. Food Protect.* 69 2777–2785. 10.4315/0362-028X-69.11.2777 17133828

[B43] Dymowska A. K. Hwang P.-P. Goss G. G. (2012). Structure and function of ionocytes in the freshwater fish gill. *Respiratory Physiol. Neurobiol.* 184 282–292. 10.1016/j.resp.2012.08.025 22981968

[B44] Epel D. Luckenbach T. Stevenson C. N. Macmanus-Spencer L. A. Hamdoun A. Smital T. (2008). Efflux Transporters: newly appreciated roles in protection against pollutants. *Environ. Sci. Technol.* 42 3914–3920. 18589945 10.1021/es087187v PMC3160781

[B45] Erickson R. J. McKim J. M. Lien G. J. Hoffman A. D. Batterman S. L. (2006). Uptake and elimination of ionizable organic chemicals at fish gills: II. Observed and predicted effects of pH, alkalinity, and chemical properties. *Environ. Toxicol. Chem.* 25 1522–1532. 10.1897/05-359R.1 16764470

[B46] Eufemia N. Clerte S. Girshick S. Epel D. (2002). Algal products as naturally occurring substrates for p-glycoprotein in Mytilus californianus. *Mar. Biol.* 140 343–353. 10.1007/s002270100693

[B47] Faria M. Navarro A. Luckenbach T. Piña B. Barata C. (2011). Characterization of the multixenobiotic resistance (MXR) mechanism in embryos and larvae of the zebra mussel (Dreissena polymorpha) and studies on its role in tolerance to single and mixture combinations of toxicants. *Aquatic Toxicol.* 101 78–87. 10.1016/j.aquatox.2010.09.004 20947180

[B48] Felmlee M. A. Jones R. S. Rodriguez-Cruz V. Follman K. E. Morris M. E. (2020). Monocarboxylate transporters (SLC16): function, regulation, and role in health and disease. *Pharmacol. Rev.* 72 466–485. 10.1124/pr.119.018762 32144120 PMC7062045

[B49] Ferreira M. Costa J. Reis-Henriques M. A. (2014). ABC transporters in fish species: a review. *Front. Physiol.* 5:266. 10.3389/fphys.2014.00266 25101003 PMC4106011

[B50] Fischer S. Klüver N. Burkhardt-Medicke K. Pietsch M. Schmidt A.-M. Wellner P. (2013). Abcb4 acts as multixenobiotic transporter and active barrier against chemical uptake in zebrafish (Danio rerio) embryos. *BMC Biol.* 11:69. 10.1186/1741-7007-11-69 23773777 PMC3765700

[B51] Foyle K. L. Hess S. Powell M. D. Herbert N. A. (2020). What is gill health and what is its role in marine finfish aquaculture in the face of a changing climate? *Front. Mar. Sci.* 7:400. 10.3389/fmars.2020.00400

[B52] Frank N. Y. Margaryan A. Huang Y. Schatton T. Waaga-Gasser A. M. Gasser M. (2005). ABCB5-mediated doxorubicin transport and chemoresistance in human malignant melanoma. *Cancer Res.* 65 4320–4333. 10.1158/0008-5472.CAN-04-3327 15899824

[B53] Fulton C. A. Huff Hartz K. E. Fuller N. W. Kent L. N. Anzalone S. E. Miller T. M. (2021). Fitness costs of pesticide resistance in Hyalella azteca under future climate change scenarios. *Sci. Total Environ.* 753:141945. 10.1016/j.scitotenv.2020.141945 32911165

[B54] Furness J. B. Cottrell J. J. Bravo D. M. (2015). Comparative gut physiology symposium: comparative physiology of digestion. *J. Anim. Sci.* 93 485–491. 10.2527/jas.2014-8481 26020739

[B55] Gabaldón T. Koonin E. V. (2013). Functional and evolutionary implications of gene orthology. *Nat. Rev. Genet.* 14 360–366. 10.1038/nrg3456 23552219 PMC5877793

[B56] Giacomini K. M. Huang S.-M. Tweedie D. J. Benet L. Z. Brouwer K. L. R. Chu X. (2010). Membrane transporters in drug development. *Nat. Rev. Drug Discov.* 9 215–236. 10.1038/nrd3028 20190787 PMC3326076

[B57] Glavinas H. Krajcsi P. Cserepes J. Sarkadi B. (2004). The role of ABC transporters in drug resistance, metabolism and toxicity. *Curr. Drug Delivery* 1 27–42. 10.2174/1567201043480036 16305368

[B58] Gordon W. E. Espinoza J. A. Leerberg D. M. Yelon D. Hamdoun A. (2019). Xenobiotic transporter activity in zebrafish embryo ionocytes. *Aquatic Toxicol.* 212 88–97. 10.1016/j.aquatox.2019.04.013 31077970 PMC6561644

[B59] Gottesman M. M. Fojo T. Bates S. E. (2002). Multidrug resistance in cancer: role of ATP-dependent transporters. *Nat. Rev. Cancer* 2 48–58. 10.1038/nrc706 11902585

[B60] Guéniche N. Bruyere A. Ringeval M. Jouan E. Huguet A. Hégarat L. L. (2020). Differential interactions of carbamate pesticides with drug transporters. *Xenobiotica* 50 1380–1392. 10.1080/00498254.2020.1771473 32421406

[B61] Guo B. Xu Z. Yan X. Buttino I. Li J. Zhou C. (2020). Novel ABCB1 and ABCC transporters are involved in the detoxification of Benzo(α)pyrene in thick shell mussel, mytilus coruscus. *Front. Mar. Sci.* 7:119. 10.3389/fmars.2020.00119

[B62] Hamdoun A. M. Cherr G. N. Roepke T. A. Epel D. (2004). Activation of multidrug efflux transporter activity at fertilization in sea urchin embryos (Strongylocentrotus purpuratus). *Dev. Biol.* 276 452–462. 10.1016/j.ydbio.2004.09.013 15581878

[B63] Harder A. M. Ardren W. R. Evans A. N. Futia M. H. Kraft C. E. Marsden J. E. (2018). Thiamine deficiency in fishes: causes, consequences, and potential solutions. *Rev. Fish Biol. Fisheries* 28 865–886. 10.1007/s11160-018-9538-x

[B64] Hasenbein S. Poynton H. Connon R. E. (2018). Contaminant exposure effects in a changing climate: how multiple stressors can multiply exposure effects in the amphipod Hyalella azteca. *Ecotoxicology (London, England)* 27 845–859. 10.1007/s10646-018-1912-x 29464532

[B65] Hediger M. A. Clémençon B. Burrier R. E. Bruford E. A. (2013). The ABCs of membrane transporters in health and disease (SLC series): introduction. *Mol. Aspects Med.* 34 95–107. 10.1016/j.mam.2012.12.009 23506860 PMC3853582

[B66] Hediger M. A. Romero M. F. Peng J.-B. Rolfs A. Takanaga H. Bruford E. A. (2004). The ABCs of solute carriers: physiological, pathological and therapeutic implications of human membrane transport proteins. *Pflügers Archiv.* 447 465–468. 10.1007/s00424-003-1192-y 14624363

[B67] Hillgren K. M. Keppler D. Zur A. A. Giacomini K. M. Stieger B. Cass C. E. (2013). Emerging transporters of clinical importance: an update from the International Transporter Consortium. *Clin. Pharmacol. Therapeutics* 94 52–63. 10.1038/clpt.2013.74 23588305

[B68] Hites R. A. Foran J. A. Carpenter D. O. Hamilton M. C. Knuth B. A. Schwager S. J. (2004a). Global assessment of organic contaminants in farmed salmon. *Science* 303 226–229. 10.1126/science.1091447 14716013

[B69] Hites R. A. Foran J. A. Schwager S. J. Knuth B. A. Hamilton M. C. Carpenter D. O. (2004b). Global assessment of polybrominated diphenyl ethers in farmed and wild salmon. *Environ. Sci. Technol.* 38 4945–4949. 10.1021/es049548m 15506184

[B70] Höglund P. J. Nordström K. J. V. Schiöth H. B. Fredriksson R. (2011). The solute carrier families have a remarkably long evolutionary history with the majority of the human families present before divergence of bilaterian species. *Mol. Biol. Evol.* 28 1531–1541. 10.1093/molbev/msq350 21186191 PMC3058773

[B71] Horio M. Gottesman M. M. Pastan I. (1988). ATP-dependent transport of vinblastine in vesicles from human multidrug-resistant cells. *Proc. Natl. Acad. Sci. U.S.A.* 85 3580–3584. 3368466 10.1073/pnas.85.10.3580 PMC280257

[B72] Hu J. Tian J. Zhang F. Wang H. Yin J. (2019). Pxr- and Nrf2- mediated induction of ABC transporters by heavy metal ions in zebrafish embryos. *Environ. Pollut.* 255:113329. 10.1016/j.envpol.2019.113329 31600704

[B73] Huang Q. Vera Delgado J. M. Seni Pinoargote O. D. Llaguno R. A. (2015). Molecular evolution of the Slc15 family and its response to waterborne copper and mercury exposure in tilapia. *Aquatic Toxicol.* 163 140–147. 10.1016/j.aquatox.2015.04.011 25897688

[B74] Hwang P.-P. Lee T.-H. Lin L.-Y. (2011). Ion regulation in fish gills: recent progress in the cellular and molecular mechanisms. *Am. J. Physiol.-Regulatory Integrat. Comparat. Physiol.* 301 R28–R47. 10.1152/ajpregu.00047.2011 21451143

[B75] Ivanis G. Esbaugh A. J. Perry S. F. (2008). Branchial expression and localization of SLC9A2 and SLC9A3 sodium/hydrogen exchangers and their possible role in acid-base regulation in freshwater rainbow trout (Oncorhynchus mykiss). *J. Exp. Biol.* 211(Pt 15) 2467–2477. 10.1242/jeb.017491 18626081

[B76] Jeffries K. M. Connon R. E. Verhille C. E. Dabruzzi T. F. Britton M. T. Durbin-Johnson B. P. (2019). Divergent transcriptomic signatures in response to salinity exposure in two populations of an estuarine fish. *Evolutionary Appl.* 12 1212–1226. 10.1111/eva.12799 31293632 PMC6597873

[B77] Jeffries K. M. Fangue N. A. Connon R. E. (2018). Multiple sub-lethal thresholds for cellular responses to thermal stressors in an estuarine fish. *Comparat. Biochem. Physiol. Part A: Mol. Integrat. Physiol.* 225 33–45. 10.1016/j.cbpa.2018.06.020 29958996

[B78] Jiang Y. Zhang S. Feng S. Sun J. Xu P. (2014). Genome wide identification, phylogeny and expression of zinc transporter genes in common carp. *PLoS One* 9:e116043. 10.1371/journal.pone.0116043 25551462 PMC4281218

[B79] Jonker J. W. Stedman C. A. M. Liddle C. Downes M. (2009). Hepatobiliary ABC transporters: physiology, regulation and implications for disease. *Front. Biosci.* 4 4904–4920. 10.2741/3576 19482594

[B80] Juliano R. L. Ling V. (1976). A surface glycoprotein modulating drug permeability in Chinese hamster ovary cell mutants. *Biochim. Biophys. Acta (BBA) - Biomembranes* 455 152–162. 10.1016/0005-2736(76)90160-7 990323

[B81] Kaler G. Truong D. M. Khandelwal A. Nagle M. Eraly S. A. Swaan P. W. (2007). Structural variation governs substrate specificity for Organic Anion Transporter (OAT) homologs: potential remote sensing by oat family members*. *J. Biol. Chem.* 282 23841–23853. 10.1074/jbc.M703467200 17553798 PMC3812435

[B82] Karasov W. H. Douglas A. E. (2013). Comparative digestive physiology. *Comprehensive Physiol.* 3 741–783. 10.1002/cphy.c110054 23720328 PMC4458075

[B83] Kiela P. R. Ghishan F. K. (2016). Physiology of intestinal absorption and secretion. *Best Pract. Res. Clin. Gastroenterol.* 30 145–159. 10.1016/j.bpg.2016.02.007 27086882 PMC4956471

[B84] Kim Y.-I. Nam I.-K. Lee D.-K. Bhandari S. Charton L. Kwak S. (2020). Slc25a17 acts as a peroxisomal coenzyme A transporter and regulates multiorgan development in zebrafish. *J. Cell. Physiol.* 235 151–165. 10.1002/jcp.28954 31187491

[B85] Komoroske L. M. Jeffries K. M. Whitehead A. Roach J. L. Britton M. Connon R. E. (2021). Transcriptional flexibility during thermal challenge corresponds with expanded thermal tolerance in an invasive compared to native fish. *Evolutionary Appl.* 14 931–949. 10.1111/eva.13172

[B86] König J. Müller F. Fromm M. F. (2013). Transporters and drug-drug interactions: important determinants of drug disposition and effects. *Pharmacol. Rev.* 65 944–966. 10.1124/pr.113.007518 23686349

[B87] Kortner T. M. Skugor S. Penn M. H. Mydland L. T. Djordjevic B. Hillestad M. (2012). Dietary soyasaponin supplementation to pea protein concentrate reveals nutrigenomic interactions underlying enteropathy in Atlantic salmon (Salmo salar). *BMC Vet. Res.* 8:101. 10.1186/1746-6148-8-101 22748053 PMC3424111

[B88] Kottra G. Daniel H. (2001). Bidirectional electrogenic transport of peptides by the proton-coupled carrier PEPT1 in Xenopus laevis oocytes: its asymmetry and symmetry. *J. Physiol.* 536 495–503. 10.1111/j.1469-7793.2001.0495c.xd 11600684 PMC2278880

[B89] Kottra G. Stamfort A. Daniel H. (2002). PEPT1 as a paradigm for membrane carriers that mediate electrogenic bidirectional transport of anionic, cationic, and neutral substrates *. *J. Biol. Chem.* 277 32683–32691. 10.1074/jbc.M204192200 12082113

[B90] Kropf C. Fent K. Fischer S. Casanova A. Segner H. (2020). ABC transporters in gills of rainbow trout (Oncorhynchus mykiss). *J. Exp. Biol.* 223:jeb221069. 10.1242/jeb.221069 32532865

[B91] Kropf C. Segner H. Fent K. (2016). ABC transporters and xenobiotic defense systems in early life stages of rainbow trout (Oncorhynchus mykiss). *Comparat. Biochem. Physiol. Part C: Toxicol. Pharmacol.* 18 45–56. 10.1016/j.cbpc.2016.02.006 26945521

[B92] Kundu G. K. Alauddin M. Akter M. S. Khan M. S. Islam M. M. Mondal G. (2017). Metal contamination of commercial fish feed and quality aspects of farmed tilapia (Oreochromis niloticus) in Bangladesh. *Biores. Commun.-(BRC)* 3 345–353.

[B93] Kurelec B. (1992). The multixenobiotic resistance mechanism in aquatic organisms. *Crit. Rev. Toxicol.* 22 23–43. 10.3109/10408449209145320 1352103

[B94] Kurelec B. (1997). A new type of hazardous chemical: the chemosensitizers of multixenobiotic resistance. *Environ. Health Perspect.* 105(Suppl. 4) 855–860. 10.1289/ehp.97105s4855 9255572 PMC1470033

[B95] Kurelec B. Lucić D. Pivćević B. Krća S. (1995). Induction and reversion of multixenobiotic resistance in the marine snail Monodonta turbinata. *Mar. Biol.* 123 305–312. 10.1007/BF00353622

[B96] Kwong R. W. M. Wang W.-X. Lam P. K. S. Yu P. K. N. (2006). The uptake, distribution and elimination of paralytic shellfish toxins in mussels and fish exposed to toxic dinoflagellates. *Aquatic Toxicol.* 80 82–91. 10.1016/j.aquatox.2006.07.016 16959334

[B97] Leslie E. M. Deeley R. G. Cole S. P. C. (2005). Multidrug resistance proteins: role of P-glycoprotein, MRP1, MRP2, and BCRP (ABCG2) in tissue defense. *Toxicol. Appl. Pharmacol.* 204 216–237. 10.1016/j.taap.2004.10.012 15845415

[B98] Li X. Dong S. Wang P. Su X. Fu J. (2019). Polychlorinated biphenyls are still alarming persistent organic pollutants in marine-origin animal feed (fishmeal). *Chemosphere* 233 355–362. 10.1016/j.chemosphere.2019.05.250 31176898

[B99] Lin L. Yee S. W. Kim R. B. Giacomini K. M. (2015). SLC transporters as therapeutic targets: emerging opportunities. *Nat. Rev. Drug Discov.* 14 543–560. 10.1038/nrd4626 26111766 PMC4698371

[B100] Ling V. (1997). Multidrug resistance: molecular mechanisms and clinical relevance. *Cancer Chemother. Pharmacol.* 40 S3–S8. 10.1007/s002800051053 9272126

[B101] Litman T. Zeuthen T. Skovsgaard T. Stein W. D. (1997). Structure-activity relationships of P-glycoprotein interacting drugs: kinetic characterization of their effects on ATPase activity. *Biochim. Biophys. Acta (BBA) - Mol. Basis Dis.* 1361 159–168. 10.1016/S0925-4439(97)00026-4 9300797

[B102] Liu F. Xia W. Hu J. Wang Y. Yang F. Sun S. (2015). Solute carrier family 26 member a2 (slc26a2) regulates otic development and hair cell survival in zebrafish. *PLoS One* 10:e0136832. 10.1371/journal.pone.0136832 26375458 PMC4573323

[B103] Liu S. Li Q. Liu Z. (2013). Genome-wide identification, characterization and phylogenetic analysis of 50 Catfish ATP-Binding Cassette (ABC) transporter genes. *PLoS One* 8:e63895. 10.1371/journal.pone.0063895 23696857 PMC3655950

[B104] Lončar J. Popović M. Krznar P. Zaja R. Smital T. (2016). The first characterization of multidrug and toxin extrusion (MATE/SLC47) proteins in zebrafish (Danio rerio). *Sci. Rep.* 6:28937. 10.1038/srep28937 27357367 PMC4928094

[B105] Lončar J. Popović M. Zaja R. Smital T. (2010). Gene expression analysis of the ABC efflux transporters in rainbow trout (Oncorhynchus mykiss). *Comparat. Biochem. Physiol. Part C: Toxicol. Pharmacol.* 151 209–215. 10.1016/j.cbpc.2009.10.009 19883795

[B106] Lončar J. Smital T. (2018). Interaction of environmental contaminants with zebrafish (*Danio rerio*) multidrug and toxin extrusion protein 7 (Mate7/Slc47a7). *Aquatic Toxicol.* 205 193–203. 10.1016/j.aquatox.2018.10.016 30396010

[B107] Long Y. Li Q. Zhong S. Wang Y. Cui Z. (2011). Molecular characterization and functions of zebrafish ABCC2 in cellular efflux of heavy metals. *Comparative Biochem. Physiol. Toxicol. Pharmacol.: CBP* 153 381–391. 10.1016/j.cbpc.2011.01.002 21266201

[B108] Love R. C. Osachoff H. L. Kennedy C. J. (2021). Short communication: tissue-specific transcript expression of P-glycoprotein isoforms abcb1a and abcb1b in rainbow trout (Oncorhynchus mykiss) following induction with clotrimazole. *Comparat. Biochem. Physiol. Part B, Biochem. Mol. Biol.* 252:110538. 10.1016/j.cbpb.2020.110538 33227421

[B109] Lu P. Liu R. Sharom F. J. (2001). Drug transport by reconstituted P-glycoprotein in proteoliposomes. *Eur. J. Biochem.* 268 1687–1697. 10.1046/j.1432-1327.2001.02041.x 11248688

[B110] Lu X. Long Y. Sun R. Zhou B. Lin L. Zhong S. (2015). Zebrafish Abcb4 is a potential efflux transporter of microcystin-LR. *Comp. Biochem. Physiol. C Toxicol. Pharmacol.* 167, 35–42. 10.1016/j.cbpc.2014.08.005 25193616

[B111] Luckenbach T. Epel D. (2005). Nitromusk and polycyclic musk compounds as long-term inhibitors of cellular xenobiotic defense systems mediated by multidrug transporters. *Environ. Health Perspect.* 113 17–24. 10.1289/ehp.7301 15626642 PMC1253704

[B112] Luckenbach T. Epel D. (2008). ABCB- and ABCC-type transporters confer multixenobiotic resistance and form an environment-tissue barrier in bivalve gills. *Am. J. Physiol.-Regulat. Integrat. Comparat. Physiol.* 294 R1919–R1929. 10.1152/ajpregu.00563.2007 18401003

[B113] Luckenbach T. Fischer S. Sturm A. (2014). Current advances on ABC drug transporters in fish. *Comparat. Biochem. Physiol. Part C: Toxicol. Pharmacol.* 165 28–52. 10.1016/j.cbpc.2014.05.002 24858718

[B114] Macdonald R. Mackay D. Hickie B. (2002). Contaminant amplification in the environment. *Environ. Sci. Technol.* 36 456A–462A. 10.1021/es022470u 12523402

[B115] Mackay D. Celsie A. K. D. Powell D. E. Parnis J. M. (2018). Bioconcentration, bioaccumulation, biomagnification and trophic magnification: a modelling perspective. *Environ. Sci.: Processes Impacts* 20 72–85. 10.1039/C7EM00485K 29260171

[B116] Mackay D. Fraser A. (2000). Bioaccumulation of persistent organic chemicals: mechanisms and models. *Environ. Pollut.* 110 375–391. 10.1016/S0269-7491(00)00162-7 15092817

[B117] Maetz J. García Romeu F. (1964). The mechanism of sodium and chloride uptake by the gills of a fresh-water fish, carassius auratus: II. Evidence for NH4+ / NA+ and HCO3- / Cl- exchanges. *J. Gen. Physiol.* 47 1209–1227. 10.1085/jgp.47.6.1209 14193849 PMC2195381

[B118] Margheritis E. Terova G. Oyadeyi A. S. Renna M. D. Cinquetti R. Peres A. (2013). Characterization of the transport of lysine-containing dipeptides by PepT1 orthologs expressed in Xenopus laevis oocytes. *Comparat. Biochem. Physiol. Part A: Mol. Integrat. Physiol.* 164 520–528. 10.1016/j.cbpa.2012.12.016 23268205

[B119] Marić P. Ahel M. Maraković N. Lončar J. Mihaljević I. Smital T. (2021). Selective interaction of microcystin congeners with zebrafish (Danio rerio) Oatp1d1 transporter. *Chemosphere* 283:131155. 10.1016/j.chemosphere.2021.131155 34182632

[B120] Martínez-Escauriaza R. Lozano V. Pérez-Parallé M. L. Blanco J. Sánchez J. L. Pazos A. J. (2021). Expression analyses of genes related to multixenobiotic resistance in mytilus galloprovincialis after exposure to okadaic acid-producing dinophysis acuminata. *Toxins* 13:614. 10.3390/toxins13090614 34564618 PMC8471661

[B121] Martins J. C. Domínguez-Pérez D. Azevedo C. Braga A. C. Costa P. R. Osório H. (2020). Molecular responses of mussel mytilus galloprovincialis associated to accumulation and depuration of marine biotoxins okadaic acid and Dinophysistoxin-1 revealed by shotgun proteomics. *Front. Mar. Sci.* 7:589822. 10.3389/fmars.2020.589822

[B122] McFadzen I. Eufemia N. Heath C. Epel D. Moore M. Lowe D. (2000). Multidrug resistance in the embryos and larvae of the mussel Mytilus edulis. *Mar. Environ. Res.* 50 319–323. 10.1016/S0141-1136(00)00057-X 11460711

[B123] Mihaljević I. Popović M. Žaja R. Maraković N. Šinko G. Smital T. (2017). Interaction between the zebrafish (Danio rerio) organic cation transporter 1 (Oct1) and endo- and xenobiotics. *Aquatic Toxicol.* 187 18–28. 10.1016/j.aquatox.2017.03.012 28363126

[B124] Mihaljevic I. Popovic M. Zaja R. Smital T. (2016). Phylogenetic, syntenic, and tissue expression analysis of slc22 genes in zebrafish (Danio rerio). *BMC Genomics* 17:626. 10.1186/s12864-016-2981-y 27519738 PMC4982206

[B125] Miller D. S. Masereeuw R. Karnaky K. J. (2002). Regulation of MRP2-mediated transport in shark rectal salt gland tubules. *Am. J. Physiol. Regulat. Integrat. Comparat. Physiol.* 282 R774–R781. 10.1152/ajpregu.00333.2001 11832398

[B126] Moreira R. Pereiro P. Canchaya C. Posada D. Figueras A. Novoa B. (2015). RNA-Seq in Mytilus galloprovincialis: comparative transcriptomics and expression profiles among different tissues. *BMC Genomics* 16:728. 10.1186/s12864-015-1817-5 26400066 PMC4581086

[B127] Müller J. Keiser M. Drozdzik M. Oswald S. (2017). Expression, regulation and function of intestinal drug transporters: an update. *Biol. Chem.* 398 175–192. 10.1515/hsz-2016-0259 27611766

[B128] Mundy P. C. Jeffries K. M. Fangue N. A. Connon R. E. (2020). Differential regulation of select osmoregulatory genes and Na+/K+-ATPase paralogs may contribute to population differences in salinity tolerance in a semi-anadromous fish. *Comparat. Biochem. Physiol. Part A: Mol. Integrat. Physiol.* 240:110584. 10.1016/j.cbpa.2019.110584 31676412 PMC7942202

[B129] Muzzio A. M. Noyes P. D. Stapleton H. M. Lema S. C. (2014). Tissue distribution and thyroid hormone effects on mRNA abundance for membrane transporters Mct8, Mct10, and organic anion-transporting polypeptides (Oatps) in a teleost fish. *Comparat. Biochem. Physiol. Part A: Mol. Integrat. Physiol.* 167 77–89. 10.1016/j.cbpa.2013.09.019 24113777 PMC4160178

[B130] Nadella S. R. Grosell M. Wood C. M. (2007). Mechanisms of dietary Cu uptake in freshwater rainbow trout: evidence for Na-assisted Cu transport and a specific metal carrier in the intestine. *J. Comparat. Physiol. B, Biochem. Syst. Environ. Physiol.* 177 433–446. 10.1007/s00360-006-0142-3 17279389

[B131] Nicklisch S. C. T. Bonito L. T. Sandin S. Hamdoun A. (2017a). Geographic differences in persistent organic pollutant levels of yellowfin tuna. *Environ. Health Perspect.* 125:067014. 10.1289/EHP518 28686554 PMC5714290

[B132] Nicklisch S. C. T. Bonito L. T. Sandin S. Hamdoun A. (2017b). Mercury levels of yellowfin tuna (Thunnus albacares) are associated with capture location. *Environ. Pollut.* 229 87–93. 10.1016/j.envpol.2017.05.070 28577385 PMC6544047

[B133] Nicklisch S. C. T. Hamdoun A. (2020). Disruption of small molecule transporter systems by Transporter-Interfering Chemicals (TICs). *FEBS Lett.* 594 4158–4185. 10.1002/1873-3468.14005 33222203 PMC8112642

[B134] Nicklisch S. C. T. Pouv A. K. Rees S. D. McGrath A. P. Chang G. Hamdoun A. (2021). Transporter-interfering chemicals inhibit P-glycoprotein of yellowfin tuna (Thunnus albacares). *Comparat. Biochem. Physiol. Part C: Toxicol. Pharmacol.* 248:109101. 10.1016/j.cbpc.2021.109101 34116183

[B135] Nicklisch S. C. T. Rees S. D. McGrath A. P. Gökirmak T. Bonito L. T. Vermeer L. M. (2016). Global marine pollutants inhibit P-glycoprotein: environmental levels, inhibitory effects, and cocrystal structure. *Sci. Adv.* 2:e1600001. 10.1126/sciadv.1600001 27152359 PMC4846432

[B136] Nigam S. K. (2015). What do drug transporters really do? *Nat. Rev. Drug Discov.* 14 29–44. 10.1038/nrd4461 25475361 PMC4750486

[B137] Nigam S. K. (2018). The SLC22 transporter family: a paradigm for the impact of drug transporters on metabolic pathways, signaling, and disease. *Annu. Rev. Pharmacol. Toxicol.* 58 663–687. 10.1146/annurev-pharmtox-010617-052713 29309257 PMC6225997

[B138] Noyes P. D. McElwee M. K. Miller H. D. Clark B. W. Van Tiem L. A. Walcott K. C. (2009). The toxicology of climate change: environmental contaminants in a warming world. *Environ. Int.* 35 971–986. 10.1016/j.envint.2009.02.006 19375165

[B139] Oosterhuis B. Vukman K. Vági E. Glavinas H. Jablonkai I. Krajcsi P. (2008). Specific interactions of chloroacetanilide herbicides with human ABC transporter proteins. *Toxicology* 248 45–51. 10.1016/j.tox.2008.03.003 18433974

[B140] Painefilú J. C. Bianchi V. A. Krock B. De Anna J. S. Kristoff G. Luquet C. M. (2020). Effects of paralytic shellfish toxins on the middle intestine of Oncorhynchus mykiss: glutathione metabolism, oxidative status, lysosomal function and ATP-binding cassette class C (ABCC) proteins activity. *Ecotoxicol. Environ. Safety* 204:111069. 10.1016/j.ecoenv.2020.111069 32758696

[B141] Pan J.-H. Feng L. Jiang W.-D. Wu P. Kuang S.-Y. Tang L. (2017). Vitamin E deficiency depressed fish growth, disease resistance, and the immunity and structural integrity of immune organs in grass carp (Ctenopharyngodon idella): referring to NF-κB, TOR and Nrf2 signaling. *Fish Shellfish Immunol.* 60 219–236. 10.1016/j.fsi.2016.11.044 27888132

[B142] Paul T. Shukla S. P. Kumar K. Poojary N. Manickavasagam S. Kumar S. (2020). Effects of temperature and pH on acute toxicity of triclosan in pangasianodon hypophthalmus (Sauvage, 1878). *Proc. Natl. Acad. Sci. India Section B: Biol. Sci.* 90 677–685. 10.1007/s40011-019-01143-4

[B143] Pizzagalli M. D. Bensimon A. Superti-Furga G. (2021). A guide to plasma membrane solute carrier proteins. *FEBS J.* 288 2784–2835. 10.1111/febs.15531 32810346 PMC8246967

[B144] Planchamp C. Hadengue A. Stieger B. Bourquin J. Vonlaufen A. Frossard J.-L. (2007). Function of both sinusoidal and canalicular transporters controls the concentration of organic anions within hepatocytes. *Mol. Pharmacol.* 71 1089–1097. 10.1124/mol.106.030759 17234897

[B145] Popovic M. Zaja R. Fent K. Smital T. (2013). Molecular characterization of zebrafish Oatp1d1 (*Slco1d1*), a novel organic anion-transporting polypeptide. *J. Biol. Chem.* 288, 33894–33911. 10.1074/jbc.M113.518506 24126916 PMC3837131

[B146] Popovic M. Zaja R. Fent K. Smital T. (2014). Interaction of environmental contaminants with zebrafish organic anion transporting polypeptide, Oatp1d1 (Slco1d1). *Toxicol. Appl. Pharmacol.* 280 149–158. 10.1016/j.taap.2014.07.015 25088042

[B147] Popovic M. Zaja R. Loncar J. Smital T. (2010b). A novel ABC transporter: the first insight into zebrafish (Danio rerio) ABCH1. *Mar. Environ. Res.* 69 S11–S13. 10.1016/j.marenvres.2009.10.016 19926124

[B148] Popovic M. Zaja R. Smital T. (2010a). Organic anion transporting polypeptides (OATP) in zebrafish (Danio rerio): phylogenetic analysis and tissue distribution. *Comparat. Biochem. Physiol. Part A, Mol. Integrat. Physiol.* 155 327–335. 10.1016/j.cbpa.2009.11.011 19931635

[B149] Protopopova M. V. Pavlichenko V. V. Luckenbach T. (2020). Changes of cellular stress response related hsp70 and abcb1 transcript and Hsp70 protein levels in Siberian freshwater amphipods upon exposure to cadmium chloride in the lethal concentration range. *PeerJ* 8:e8635. 10.7717/peerj.8635 32195047 PMC7067181

[B150] Ray A. K. Ringø E. (2014). “The gastrointestinal tract of fish,” in *Aquaculture Nutrition*, eds Merrifield D. Ringo E. (Hoboken, NJ: John Wiley & Sons, Ltd), 1–13.

[B151] Riisgård H. U. Egede P. P. Barreiro Saavedra I. (2011). Feeding behaviour of the mussel, mytilus edulis: new observations, with a minireview of current knowledge. *J. Mar. Biol.* 2011:e312459. 10.1155/2011/312459

[B152] Riisgård H. U. Funch P. Larsen P. S. (2015). The mussel filter–pump – present understanding, with a re-examination of gill preparations. *Acta Zool.* 96 273–282. 10.1111/azo.12110

[B153] Rimoldi S. Bossi E. Harpaz S. Cattaneo A. G. Bernardini G. Saroglia M. (2015). Intestinal B0AT1 (SLC6A19) and PEPT1 (SLC15A1) mRNA levels in European sea bass (Dicentrarchus labrax) reared in fresh water and fed fish and plant protein sources. *J. Nutr. Sci.* 4:e21. 10.1017/jns.2015.9 26097704 PMC4462763

[B154] Romano A. Barca A. Storelli C. Verri T. (2014). Teleost fish models in membrane transport research: the PEPT1(SLC15A1) H+–oligopeptide transporter as a case study. *J. Physiol.* 592(Pt 5) 881–897. 10.1113/jphysiol.2013.259622 23981715 PMC3948553

[B155] Romano A. Kottra G. Barca A. Tiso N. Maffia M. Argenton F. (2006). High-affinity peptide transporter PEPT2 (SLC15A2) of the zebrafish Danio rerio: functional properties, genomic organization, and expression analysis. *Physiol. Genomics* 24 207–217. 10.1152/physiolgenomics.00227.2005 16317081

[B156] Romney A. L. T. Yanagitsuru Y. R. Mundy P. C. Fangue N. A. Hung T.-C. Brander S. M. (2019). Developmental staging and salinity tolerance in embryos of the delta smelt, Hypomesus transpacificus. *Aquaculture* 511:634191. 10.1016/j.aquaculture.2019.06.005 32831418 PMC7442155

[B157] Ross E. Behringer D. (2019). Changes in temperature, pH, and salinity affect the sheltering responses of Caribbean spiny lobsters to chemosensory cues. *Sci. Rep.* 9:4375. 10.1038/s41598-019-40832-y 30867504 PMC6416250

[B158] Said H. M. Nexo E. (2018). “Chapter 54 - intestinal absorption of water-soluble vitamins: cellular and molecular mechanisms,” in *Physiology of the Gastrointestinal Tract*, 6th Edn, ed. Said H. M. (Cambridge, MA: Academic Press), 1201–1248.

[B159] Schlessinger A. Yee S. W. Sali A. Giacomini K. M. (2013). SLC classification: an update. *Clin. Pharmacol. Therapeutics* 94 19–23. 10.1038/clpt.2013.73 23778706 PMC4068830

[B160] Schoels M. Zhuang M. Fahrner A. Küchlin S. Sagar, Franz H. (2021). Single-cell mRNA profiling reveals changes in solute carrier expression and suggests a metabolic switch during zebrafish pronephros development. *Am. J. Physiol.-Renal Physiol.* 320 F826–F837. 10.1152/ajprenal.00610.2020 33749326

[B161] Segarra A. Mauduit F. Amer N. R. Biefel F. Hladik M. L. Connon R. E. (2021). Salinity changes the dynamics of pyrethroid toxicity in terms of behavioral effects on newly hatched delta smelt larvae. *Toxics* 9:40. 10.3390/toxics9020040 33672739 PMC7924609

[B162] Sharom F. J. (2008). ABC multidrug transporters: structure, function and role in chemoresistance. *Pharmacogenomics* 9 105–127. 10.2217/14622416.9.1.105 18154452

[B163] Smital T. Kurelec B. (1998). The chemosensitizers of multixenobiotic resistance mechanism in aquatic invertebrates: a new class of pollutants. *Mutation Res.* 399 43–53. 10.1016/s0027-5107(97)00265-0 9635488

[B164] Smital T. Luckenbach T. Sauerborn R. Hamdoun A. M. Vega R. L. Epel D. (2004). Emerging contaminants—pesticides, PPCPs, microbial degradation products and natural substances as inhibitors of multixenobiotic defense in aquatic organisms. *Mutat. Res.* 552, 101–117. 10.1016/j.mrfmmm.2004.06.006 15288544

[B165] Smital T. Sauerborn R. (2002). Measurement of the activity of multixenobiotic resistance mechanism in the common carp Cyprinus carpio. *Mar. Environ. Res.* 54 449–453. 10.1016/S0141-1136(02)00155-1 12408600

[B166] Smital T. Sauerborn R. Pivcević B. Krca S. Kurelec B. (2000). Interspecies differences in P-glycoprotein mediated activity of multixenobiotic resistance mechanism in several marine and freshwater invertebrates. *Comparat. Biochem. Physiol. Toxicol. Pharmacol.: CBP* 126 175–186. 10.1016/s0742-8413(00)00110-9 11050689

[B167] Song F. Xu D. Zhou H. Xu W. Mai K. He G. (2017). The differences in postprandial free amino acid concentrations and the gene expression of PepT1 and amino acid transporters after fishmeal partial replacement by meat and bone meal in juvenile turbot (Scophthalmus maximus L.). *Aquaculture Res.* 48 3766–3781. 10.1111/are.13203

[B168] Song W. Li D. Tao L. Luo Q. Chen L. (2020). Solute carrier transporters: the metabolic gatekeepers of immune cells. *Acta Pharmaceutica Sin. B* 10 61–78. 10.1016/j.apsb.2019.12.006 31993307 PMC6977534

[B169] Staud F. Cerveny L. Ahmadimoghaddam D. Ceckova M. (2013). Multidrug and toxin extrusion proteins (MATE/SLC47); role in pharmacokinetics. *Int. J. Biochem. Cell Biol.* 45 2007–2011. 10.1016/j.biocel.2013.06.022 23831838

[B170] Stevenson C. N. MacManus-Spencer L. A. Luckenbach T. Luthy R. G. Epel D. (2006). New perspectives on perfluorochemical ecotoxicology: inhibition and induction of an efflux transporter in the marine mussel, mytilus californianus. *Environ. Sci. Technol.* 40 5580–5585. 10.1021/es0602593 16999143

[B171] Streit B. (1998). “Bioaccumulation of contaminants in fish,” in *Fish Ecotoxicology*, eds Braunbeck T. Hinton D. E. Streit B. (Basel: Birkhäuser).

[B172] Sundell K. S. Rønnestad I. (2011). “integrated function and control of the gut | intestinal absorption,” in *Encyclopedia of Fish Physiology*, ed. Farrell A. P. (Cambridge, MA: Academic Press), 1311–1321.

[B173] Takesono A. Moger J. Farooq S. Cartwright E. Dawid I. B. Wilson S. W. (2012). Solute carrier family 3 member 2 (Slc3a2) controls yolk syncytial layer (YSL) formation by regulating microtubule networks in the zebrafish embryo. *Proc. Natl. Acad. Sci. U.S.A.* 109 3371–3376. 10.1073/pnas.1200642109 22331904 PMC3295254

[B174] Talukder Shefat S. H. (2018). Nutritional fish disease and public health concern. *Poultry Fisheries Wildlife Sci.* 6:199. 10.4172/2375-446X.1000199

[B175] Tian J. Hu J. Liu G. Yin H. Chen M. Miao P. (2019). Altered Gene expression of ABC transporters, nuclear receptors and oxidative stress signaling in zebrafish embryos exposed to CdTe quantum dots. *Environ. Pollut.* 244 588–599. 10.1016/j.envpol.2018.10.092 30384064

[B176] To V. P. T. H. Masagounder K. Loewen M. E. (2019). SLC transporters ASCT2, B0AT1-like, y+LAT1, and LAT4-like associate with methionine electrogenic and radio-isotope flux kinetics in rainbow trout intestine. *Physiol. Rep.* 7:e14274. 10.14814/phy2.14274 31705630 PMC6841986

[B177] Truong D. M. Kaler G. Khandelwal A. Swaan P. W. Nigam S. K. (2008). Multi-level analysis of organic anion transporters 1, 3, and 6 reveals major differences in structural determinants of antiviral discrimination*. *J. Biol. Chem.* 283 8654–8663. 10.1074/jbc.M708615200 18174163 PMC2417182

[B178] Vacca F. Barca A. Gomes A. S. Mazzei A. Piccinni B. Cinquetti R. (2019). The peptide transporter 1a of the zebrafish Danio rerio, an emerging model in nutrigenomics and nutrition research: molecular characterization, functional properties, and expression analysis. *Genes Nutr.* 14:33. 10.1186/s12263-019-0657-3 31890051 PMC6923934

[B179] Verri T. Barca A. Pisani P. Piccinni B. Storelli C. Romano A. (2017). Di- and tripeptide transport in vertebrates: the contribution of teleost fish models. *J. Comparat. Physiol. B* 187 395–462. 10.1007/s00360-016-1044-7 27803975

[B180] Verri T. Romano A. Barca A. Kottra G. Daniel H. Storelli C. (2010). Transport of di- and tripeptides in teleost fish intestine. *Aquaculture Res.* 41 641–653. 10.1111/j.1365-2109.2009.02270.x

[B181] Verri T. Terova G. Romano A. Barca A. Pisani P. Storelli C. (2012). “The SoLute Carrier (SLC) family series in teleost fish,” in *Functional Genomics in Aquaculture*, eds Saroglia M. Liu Z. (Hoboken, NJ: John Wiley & Sons, Ltd), 219–320.

[B182] Wang K. Jiang W. Wu P. Liu Y. Jiang J. Kuang S. (2018). Gossypol reduced the intestinal amino acid absorption capacity of young grass carp (Ctenopharyngodon idella). *Aquaculture* 492 46–58. 10.1016/j.aquaculture.2018.03.061

[B183] Wang K. Wang E. Qin Z. Zhou Z. Geng Y. Chen D. (2016). Effects of dietary vitamin E deficiency on systematic pathological changes and oxidative stress in fish. *Oncotarget* 7 83869–83879. 10.18632/oncotarget.13729 27911874 PMC5356631

[B184] Wang X. Wang W.-X. (2015). Physiologically based pharmacokinetic model for inorganic and methylmercury in a marine fish. *Environ. Sci. Technol.* 49 10173–10181. 10.1021/acs.est.5b02301 26214348

[B185] Winter T. N. Elmquist W. F. Fairbanks C. A. (2011). OCT2 and MATE1 provide bidirectional agmatine transport. *Mol. Pharmaceutics* 8 133–142. 10.1021/mp100180a 21128598 PMC4589871

[B186] Wu W. Dnyanmote A. V. Nigam S. K. (2011). Remote communication through solute carriers and ATP binding cassette drug transporter pathways: an update on the remote sensing and signaling hypothesis. *Mol. Pharmacol.* 79 795–805. 10.1124/mol.110.070607 21325265 PMC3082935

[B187] Yan G. Zhang Y. Yu J. Yu Y. Zhang F. Zhang Z. (2012). Slc39a7/zip7 plays a critical role in development and zinc homeostasis in zebrafish. *PLoS One* 7:e42939. 10.1371/journal.pone.0042939 22912764 PMC3418240

[B188] Zaja R. Lončar J. Popovic M. Smital T. (2011). First characterization of fish P-glycoprotein (abcb1) substrate specificity using determinations of its ATPase activity and calcein-AM assay with PLHC-1/dox cell line. *Aquat. Toxicol.* 103, 53–62. 10.1016/j.aquatox.2011.02.005 21392495

[B189] Zaja R. Popović M. Lončar J. Smital T. (2016). Functional characterization of rainbow trout (*Oncorhynchus mykiss*) Abcg2a (Bcrp) transporter. *Comp. Biochem. Physiol. Toxicol. Pharmacol. CBP*, 190, 15–23. 10.1016/j.cbpc.2016.07.005 27475308

[B190] Zhang Q.-L. Dong Z.-X. Luo Z.-W. Zhang M. Deng X.-Y. Guo J. (2020). The impact of mercury on the genome-wide transcription profile of zebrafish intestine. *J. Hazardous Mater.* 389:121842. 10.1016/j.jhazmat.2019.121842 31879112

[B191] Zhang T. Yao J. Xu D. Ma X. Jin W. Lv G. (2021). Gill physiological and transcriptomic response of the threatened freshwater mussel Solenaia oleivora to salinity shift. *Comparat. Biochem. Physiol. Part D, Genomics Proteomics* 40:100913. 10.1016/j.cbd.2021.100913 34662852

[B192] Zhang Y. Zhang Y. Sun K. Meng Z. Chen L. (2018). The SLC transporter in nutrient and metabolic sensing, regulation, and drug development. *J. Mol. Cell Biol.* 11 1–13. 10.1093/jmcb/mjy052 30239845 PMC6359923

[B193] Zucchi S. Corsi I. Luckenbach T. Bard S. M. Regoli F. Focardi S. (2010). Identification of five partial ABC genes in the liver of the Antarctic fish Trematomus bernacchii and sensitivity of ABCB1 and ABCC2 to Cd exposure. *Environ. Pollut.* 158 2746–2756. 10.1016/j.envpol.2010.04.012 20627496

